# Understanding the Emergence of Ebola Virus Disease in Sierra Leone: Stalking the Virus in the Threatening Wake of Emergence

**DOI:** 10.1371/currents.outbreaks.9a6530ab7bb9096b34143230ab01cdef

**Published:** 2015-04-20

**Authors:** Nadia Wauquier, James Bangura, Lina Moses, Sheik Humarr Khan, Moinya Coomber, Victor Lungay, Michael Gbakie, Mohammed S.K. Sesay, Ibrahim A.K. Gassama, James L.B. Massally, Aiah Gbakima, James Squire, Mohamed Lamin, Lansana Kanneh, Mohammed Yillah, Kandeh Kargbo, Willie Roberts, Mohammed Vandi, David Kargbo, Tom Vincent, Amara Jambai, Mary Guttieri, Joseph Fair, Marc Souris, Jean Paul Gonzalez

**Affiliations:** Sorbonne Université, UPMC, Paris, France; Metabiota Inc., Freetown, Sierra Leone; Tulane University, New Orleans, Louisiana, USA; Ministry of Heath and Sanitation, Sierra Leone; Metabiota Inc., Kenema Government Hospital, Kenema, Sierra Leone; Metabiota Inc., Freetown, Sierra Leone; Ministry of Heath and Sanitation, Sierra Leone; Tulane University, New Orleans, Louisiana, USA; Ministry of Heath and Sanitation, Sierra Leone; Ministry of Heath and Sanitation, Sierra Leone; Ministry of Heath and Sanitation, Sierra Leone; Ministry of Heath and Sanitation, Sierra Leone; Metabiota Inc., Freetown, Sierra Leone; Ministry of Heath and Sanitation, Sierra Leone; Ministry of Heath and Sanitation, Sierra Leone; Tulane University, New Orleans, Louisiana, USA; Ministry of Heath and Sanitation, Sierra Leone; Metabiota Inc., Freetown, Sierra Leone; Metabiota Inc., Freetown, Sierra Leone; Ministry of Heath and Sanitation, Sierra Leone; Ministry of Heath and Sanitation, Sierra Leone; Directorate of Disease Prevention and Control, DPC Ministry of Health and Sanitation, Freetown, Sierra Leone; Metabiota Inc., Silver Spring and San Francisco, USA; Ministry of Heath and Sanitation, Sierra Leone; Metabiota Inc., Silver Spring and San Francisco, USA; Fondation Mérieux USA, Washington DC, USA; UMRD 190 Emergence des Pathologies Virales, IRD, Aix-Marseille University, Vientiane, Laos; Metabiota Inc., Silver Spring and San Francisco, USA

**Keywords:** Ebola Virus Disease, emergence, index case, Sierra Leone

## Abstract

Since Ebola Virus Disease (EVD) was first identified in 1976 in what is now the Democratic Republic of Congo, and despite the numerous outbreaks recorded to date, rarely has an epidemic origin been identified. Indeed, among the twenty-one most documented EVD outbreaks in Africa, an index case has been identified four times, and hypothesized in only two other instances. The initial steps of emergence and spread of a virus are critical in the development of a potential outbreak and need to be thoroughly dissected and understood in order to improve on preventative strategies. In the current West African outbreak of EVD, a unique index case has been identified, pinpointing the geographical origin of the epidemic in Guinea. Herein, we provide an accounting of events that serve as the footprint of EVD emergence in Sierra Leone and a road map for risk mitigation fueled by lessons learned.

## Introduction

Ebola Virus Disease (EVD) was first medically recorded in 1976 when the virus emerged in what is now the Democratic Republic of Congo. The identification of this new disease, which presented similarly to that of Marburg Disease, led to the recognition of Viral Hemorrhagic Fevers (VHF) as a nosological entity. Despite the many studies conducted on EVD to date, rarely has the epidemic origin (the primary infectious event) been identified. Indeed, among the twenty-one most documented outbreaks of EVD in Africa, an index case was identified four times and hypothesized in two other instances^1^^,^ 2 ^,^ 3 ^,^ 4 ^,^ 5 ^,^ 6 .

The difficulty of pinpointing the ports of viral entry into the human population mainly relies on the fact that these outbreaks often occur in remote regions that lack experienced epidemiologists which lead to delayed and unsuccessful investigations. Given the complexity of the task, it is remarkable that, in the current West African outbreak, a unique index case has been identified, defining with near certainty the geographic origin of the epidemic in Guinea. Identification of the first infected human was the result of intensive forensic work performed by a multidisciplinary team, which acted quickly to address the emergency during the initial onset of the epidemic^2^.

While apprehending the mechanisms of emergence of a zoonotic virus from an animal host to humans is critical to develop preventive strategies, a deep understanding of the immediate progression within the human population to the point of recognition by the local public health system is essential to improve on national surveillance and accelerate detection and thereby response to a nascent epidemic. Invaluable lessons can be drawn through thorough dissection of the early events, when an outbreak runs unnoticed by the health system. Ascertaining the initial spread of the virus from one human host to another is therefore critical to identify strategies to improve future outbreak response efforts.

Herein, we provide an accounting of events that serve as the footprint of EVD emergence in Sierra Leone.

## Methods

**Cases and Epidemiological Investigations**

Patients that met the World Health Organization (WHO) case definitions for suspected or probable EVD^7^ were investigated by District Health Medical Teams (DHMT) and investigation teams led by the Sierra Leone Ministry of Health and Sanitation (MOHS) and supported by international partners. Demographic, geographic, epidemiological, and clinical data were recorded on standard case investigation forms delivered by the WHO. Source of infection of each case was sought by tracing contacts retrospectively.

**Sample Collection**

Suspected or probable EVD cases were sampled, when possible, directly in the field by the DHMT or at various health-care and holding centers by clinical teams. Venous blood was drawn into collection tubes with or without EDTA, and oral swabs were collected on corpses and placed in viral transport medium (Σ-Virocult, Medical Wire). All samples were transported directly to the VHF laboratory (formerly Lassa fever Laboratory) located within the Kenema Government Hospital (KGH), for immediate testing.

**Emergency Diagnostics**

As part of the National Response Plan to the EVD outbreak in Sierra Leone^8^, and with guidance from the Sierra Leone MOHS and the WHO, emergency diagnostics were performed at the VHF Laboratory. All blood samples collected from suspected and probable cases throughout Sierra Leone between March 22 and July 2 and most blood samples collected from July 2 to August 22, were tested by the VHF laboratory. Starting July 2, Public Health Agency Canada set up a mobile laboratory in Kailahun town, Kailahun District to further support diagnostic efforts. Eventually, in late August, a Centers for Disease Control and Prevention (CDC) mobile laboratory was set up at KGH, and took over EVD diagnostics as of 22nd August. All handling and testing of samples in the VHF laboratory was performed in full BSL3 level personnel protective equipment including face, eye and respiratory protection.

**Quantitative Real-time RT-PCR**

RNA was extracted from 140μl serum using RNAeasy kits from Qiagen (Venlo, Limburg) in a Class II Biosafety cabinet. Extracts were immediately tested for viral RNA using published and FDA-approved protocols and reagents from the Critical Reagents Program and a Roche (Basel, Switzerland) Lightcyler, software version 2.0. Capillaries contained 14.6μl Ebola Zaire Master Mix, 0.4 Taq polymerase, and 5μl sample. Cycling conditions were as follows: 50°C for 15 minutes (1 cycle), 95°C for 5 minutes (1 cycle), 95°C for 1 second and 60°C for 20 seconds with a single acquisition (45 cycles), 40°C for 30 seconds (1 cycle). Samples were tested in duplicate, including an Ebola Zaire HPLC RNA positive control and a negative (mastermix alone) control in each run.

**Ethics**

The need for written informed consent was waived by the MOHS, Sierra Leone, in the context of an emergency response to an ongoing EVD outbreak. The study performed here was approved by Western International Review Board (WIRB) and Sierra Leone Ethics and Scientific Review Committee.

## Results

**Figure S1. Administrative map of the Districts of Sierra Leone.**
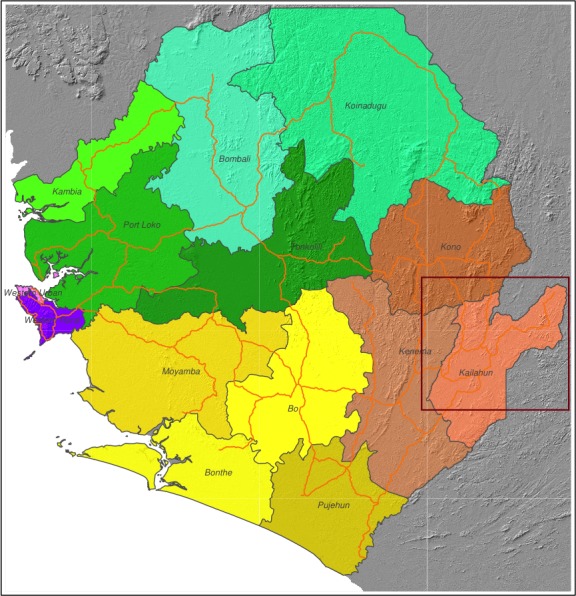
Green = Northern province districts (including Ebola Virus Disease epidemic districts of Port Loko and Kambia among others); Purple = Western areas districts; Yellow = Southern Province districts; Orange = Eastern province districts (including main Ebola Virus Disease epidemic of Kailahun and Kenema districts); Black line = district delineation; Orange line = main roads. Red square = Study area of Ebola Virus Disease emergence in country.

For centuries, Kissi people have lived in the Kissidougou region that extends across Guinea, Liberia, and Sierra Leone, which are divided by administrative borders inherited from colonial times. The Kissi inhabitants have historically travelled throughout this region, visiting community members to support their needs: attending births, marriages, and burial ceremonies. It is here, in the Kissidougou territory that the first EVD epidemic chains burst in Guinea. During this initial stage, the Kissi community followed their traditions in earnest, burying their dead, intimately supporting struggling families and patients, and seeking help from nearby traditional healers. In this context, a few kilometers away from the emerging epidemic in Guékédou (Guinea), infected individuals crossed the border to Sierra Leone (Figure S1, S2).

**Fig 1B. Ebola Virus Fever (EVD) emergence in Sierra Leone.**
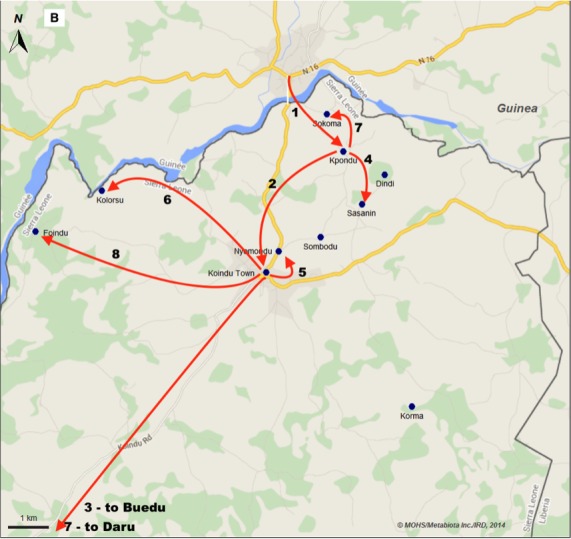
The first villages (blue points) form the North Eastern part of the Kailahun District that reported confirmed case of EVD and become EVD epidemic chain of transmission. Red arrow = form 1 to 8 showing the chronology (numbers) and direction (arrow) of the spread of the EVD during the two first week of the epidemic.

A traditional healer from the village of Kpondu, Kailahun District in Sierra Leone treated patients arriving from Guinea and Liberia that sought medical assistance. (Figure 1A,B). On April 28, the healer became extremely ill and died two days later. Several days later, in early May, two close relatives living in the same household as the healer (her husband and grandson) also died in Kpondu. Ultimately, most of those who attended her funeral ceremony became sick, each infected with the yet-to-be-identified Ebola virus.

**A. Ebola Virus Fever (EVD) emergence in Sierra Leone:**
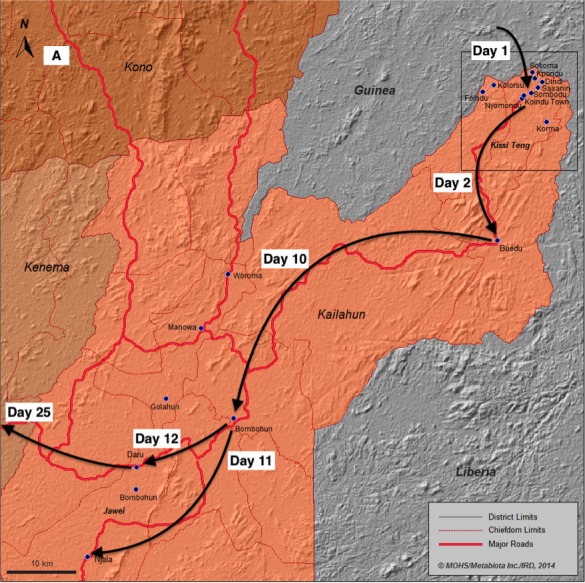
The Kailahun District (Orange) that has been the first one reporting confirmed cases of EVD in country. Villages and town reporting EVD laboratory confirmed cases: Blue point; main road = red line; administrative boundaries = grey line. Black arrow = Chronology and direction of the EVD spread. Top right square delineate the emergence zone (see 1B) where intense transmission occurred for day 1 to day 21 after confirmation of the index case.

Medical officers in the border chiefdoms of Sierra Leone had been undergoing preparedness training since the first cases occurred in Guinea and were aware of the risk of introduction of EVD into the country. It was in the small town of Koindu, five miles away and within walking distance of Kpondu (approximately three miles from the Guinean border), that the first suspected cases of EVD came to the attention of local health authorities. On May 24^th^, the Community Health Officer at Koindu Health Center notified the Kailahun District Health Management Team of three patients, who presented with fever, vomiting, and diarrhea. Each had attended the healer’s funeral. The medical staff in charge at the Center managed the patients for gastroenteritis and, suspecting a resurgence of cholera, sent stool samples to Freetown for diagnostics. Taking into consideration the ongoing and nearby epidemic of EVD, the Community Health Officer also contacted the in-country lead of the Lassa Fever Project at the Kenema Government Hospital (KGH), the late Dr. Sheik Humarr Khan. Blood samples were sent immediately to Kenema for molecular testing at the VHF Laboratory. One of the initial three patients, a 42-year old housewife, became the first laboratory-confirmed case of EVD in Sierra Leone and later, the first Sierra Leonean to survive Ebola. Ultimately, fourteen individuals who attended the burial of the traditional healer contracted the disease.

The VHF Laboratory at KGH, a regional reference center for Lassa fever diagnostics in West Africa and a base of operations for collaborative research, was the only laboratory in Sierra Leone with the capacity to test for EVD. Beginning with the emergence of EVD in Guinea, samples from VHF-suspected cases throughout Sierra Leone were sent to this laboratory for testing. On the afternoon of May 25^th^, the laboratory received the first blood sample from Koindu. Using reagents provided by the US Critical Reagents Program (CRP) in coordination with the US Army Medical Research Institute of Infectious Diseases (USAMRIID), the sample was analyzed by real-time reverse transcription polymerase chain reaction (RT-PCR), along with a batch of other routine samples received that same day. Testing confirmed that the woman in Koindu was infected with Ebola virus, a finding that was immediately reported by Dr. Khan. Testing also confirmed that two other women were infected, both of whom were patients at KGH, one admitted on the Annex Ward and the other on the Maternity Ward. The latter patient had undergone a spontaneous abortion and later became the first Ebola survivor discharged from KGH. Both cases were confirmed positive the following day using an antigen-capture enzyme-linked immune-sorbent assay (ELISA) with USAMRIID reagents, and both were isolated on the Lassa ward. Subsequently, all KGH healthcare workers who had been in contact with these women were closely monitored.

**Figure S3. Northern Kailahun District (Eastern Province of Sierra Leone) where Ebola Virus Disease emerged in country on May 25, 2014.**
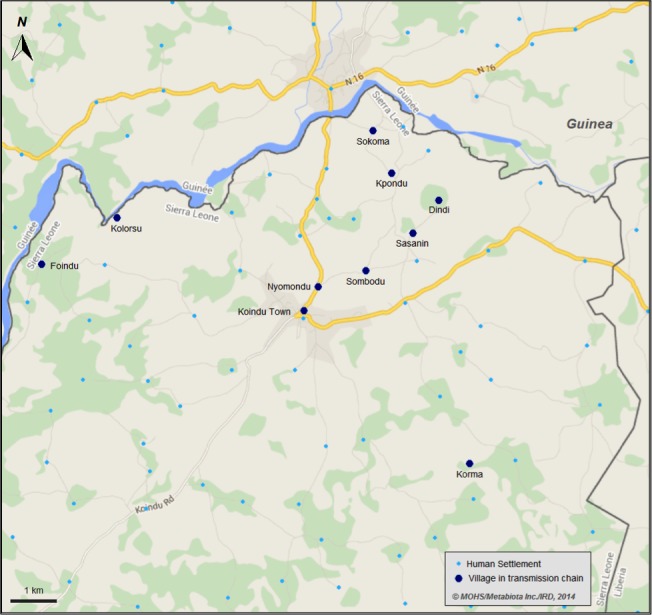
Dark blue solid circle = Villages with epidemic chain of transmission during the 3 first week of EVD emergence in country (May to June 2014); Light blue solid circle = Villages without epidemic chain; Yellow line = main roads; Simple and double gray lines = secondary roads

On May 26th, the doctor in charge of the Lassa ward immediately sent an investigation team to Koindu and surrounding villages on the border of Guinea to inform local health authorities of the risk, determine the geographical origin of the laboratory-confirmed EVD cases, identify contacts, organize training for case management, isolate suspected cases, and investigate reported deaths. Together with Kailahun district health officials and surveillance officers, the outreach team initiated their investigation in Koindu, then rapidly expanded their efforts to include Kpondu, Kolorsu, Sasani, and Nyumondu, villages of the Kissi Tenge Chiefdom (Kailahun District) (Fig 1; Fig.S2; Fig.S3).

Through May 26^th^, of twelve suspected EVD cases investigated since the first case in Koindu, seven tested positive for EVD. By May 27^th^, it was clear that transmission chains had started in the nearby villages of Buedu, Nyumudu, and Kolorsu, while two other localities, Fokoma and Kpondu, reported their first laboratory-confirmed cases of EVD. In less than four weeks, five villages and the towns of Koindu and Buedu were found affected by EVD. During this early phase of disease transmission, great fear of increasing fatalities (case fatality rate of 1:2) combined with lack of communication and information in the most remote Kissi communities led to the development of social unrest. Ultimately, a few families forcefully retrieved their sick relatives from the health center, performed high-risk traditional burial ceremonies and denied access of the responding teams to affected villages.

One of the nurses who had tended to the infected patients at Koindu Health Center became ill on May 18th. She set out for KGH to seek treatment, traveling from Koindu to Daru via public transport. Too sick to continue, she stopped at the Daru Community Health Center (CHC) where she died on the 24th of May. Her corpse was handed over to the family for traditional burial in Njala village (Jawei Chiefdom), a few kilometers away. Three health workers who admitted and treated her at Daru CHC became ill and died, including the Community Health Officer in charge. Infected local health workers and their families in Daru, together with those who participated in the nurse’s burial ceremony, sparked two new fast-growing epidemic chains in the surrounding villages of Njala and Bumbuhun. Meanwhile, the driver, who had taken the nurse from Koindu to Daru, returned to his residence in Kambia District, in the northwestern part of the country more than 500km from Koindu, first stopping in Masiaka of Port Loko District. On May 29^th^, he also developed symptoms and infected two relatives in Masiaka. Rapid response in Port Loko and Kambia led to the isolation of these three cases, and no further transmission events occurred.

At this point in time, 35 days after the death of the traditional healer, there were 30 confirmed cases from Kailahun District, five active epidemic chains, and sporadic cases were being identified in another district, Port Loko. Two weeks later, on June 17^th^, the first laboratory-confirmed case in Kenema district was identified: a 40-year old female health worker from the Kenema Township Burma II section, Nongowa Chiefdom. She had been in contact with a nurse from Golahun in Kailahun district who had participated in the funeral ceremony of the nurse in Njala. She became ill on June 7^th^and travelled from Kailahun to Kenema where she was admitted to the Female Ward at KGH. While on ward rounds, Dr. Khan identified her as an EVD-suspected case, and on June 10^th^, testing revealed she was infected. A male nurse, who had assisted in caring for her in Kenema, became sick and, on June 19^th^, also tested positive. Neither of these nurses survived (Fig. 2.).

**Ebola Virus Disease emergence in Sierra Leone, 2014: Main unprecedented epidemic chain by chiefdom, Kailahun District.**
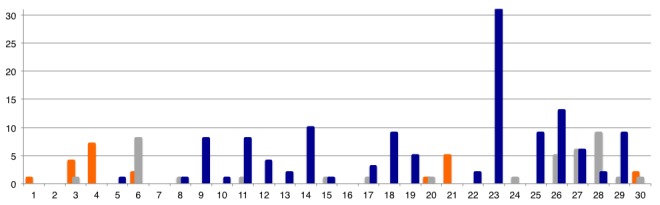
Ordinate = Number of case; abscise = Days; Bar = Three major epidemic chains that’s sparked the outbreak in country (Kissi Tengue = orange bar; Nongowa = Black bar; Jawei = Dark blue bar). Starting on day 1 as for the 25th of Mai 2014 when the first EVD case was confirmed form Kissi Tengue chiefdom. For each chiefdom, one can clearly observed two waves of cases: the second wave constituted by secondary contacts from the first wave (i.e. emerging case in the chiefdom territory) with an estimated incubation period of 10+/- 5 days between the two waves.

Kenema District rapidly became the third epicenter of the expanding Ebola outbreak in Sierra Leone. From the case of the traditional healer, the disease spread within four days to eleven individuals in the same chiefdom and in less than a week, to twenty-five cases in the contiguous Jawei chiefdom. Twenty days after the index case in Sierra Leone, eight chiefdoms of the Kailahun district displayed active epidemic chains. Six weeks after EVD emergence in Koindu, three districts were severely affected by the EVD epidemic, including Kailahun, Kenema, and Port Loko. The exponential start of the Sierra Leone outbreak was insidious and rapid. Within less than six weeks, the national healthcare system was overwhelmed, and the virus continued to replicate amongst naïve populations.

## Discussion

**Figure S4. The spread of Ebola Virus Disease (EVD) during the first phase of its extension in Sierra Leone.**
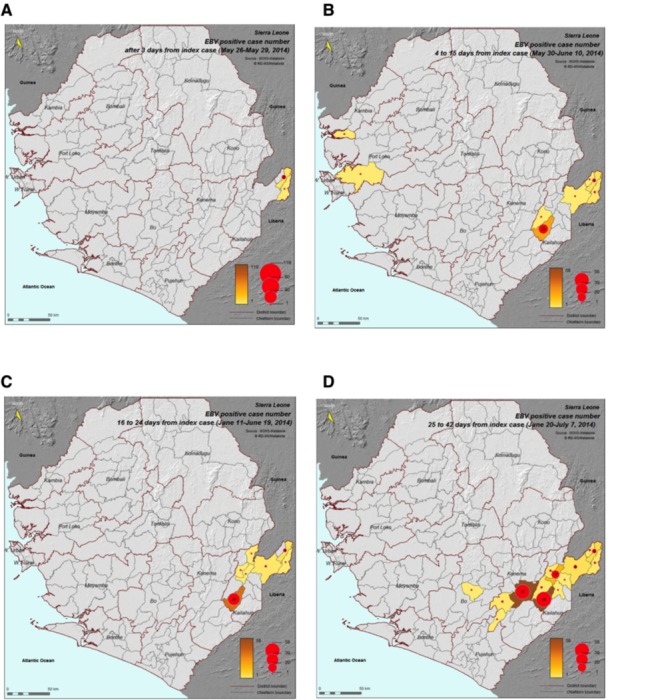
Laboratory cumulative confirmed EVD case by chiefdom for four consecutive period of time during the first phase of EVD epidemic in Sierra Leone (2014). Left to right, top to bottom: (A) May 26 to May 29; (B) May 30 to June 10. (C) June 11 to June 19. (D) June 20 to July 7.

These events make up the beginning of the EVD outbreak in Sierra Leone, the third major step of the virus, after Guinea and Liberia, towards developing the largest EVD epidemic in history. The identification on May 25^th^of the first confirmed case of EVD in Sierra Leone prompted local health authorities to immediately conduct extensive investigations, searching for suspected cases and to inform and engage the international community such as the WHO or Medecins Sans Frontières (MSF) in all response activities. Despite previous in-country experience with Lassa fever, a rapid response by well-informed field teams, availability of timely and effective laboratory diagnostics, and immediate isolation of infected individuals, expansion of the outbreak did not relent (Fig. S4). Retrospective investigations show that the virus had been circulating for at least four weeks and had spread through traditional burials and traditional healthcare settings, activities that are well known amplifiers of EVD outbreaks. Furthermore, high mobility of the infected contacts led to a rapid geographic expansion within Sierra Leone, which divided the response forces on the ground. The resulting exponential increase in case numbers rapidly overwhelmed the limited number of healthcare workers and adequate health facilities. Collectively, these factors created what is publicly referenced as the perfect storm, allowing the virus to thrive and cross new borders.^7^

Investigations are continuing to improve our understanding of the onset of the outbreak in Sierra Leone and other factors explaining the sudden and rapid increase in case numbers have not been ruled out. The Ebola community is looking into the possible existence of transmission events, prior to the infection of the traditional healer, which would imply that the virus was circulating unnoticed in Sierra Leone for a longer period of time. Additionally, a potential second independent and simultaneous introduction of EVD is currently under investigation (personal communications between authors, March 2015). Indeed, reports of a probable case that had returned sick from Guinea and a subsequent epidemic chain that developed in early May originating in Manowa village, Kailahun District, are to be dissected.

The current outbreak confirms yet again the critical role of and risk incurred by healthcare workers. The death toll of West African physicians, nurses, community health officers and other supporting staff is immense. Very rapidly in the outbreak, Sierra Leone suffered the loss of the healthcare workers leading the response activities.^9^ Protection of healthcare workers is ever more paramount as these individuals serve such a key role in ending the outbreak. Major efforts to distribute the appropriate skills and tools through infection prevention and control training and use of personnel protective equipment, need to be continued to ensure that healthcare personnel abide by the classic rules of sanitary security enforced by international responding agencies.^10^ Adding to the disease burden, is insufficient medical coverage and access to healthcare in the low resource setting of West Africa. Health posts serve a role as sentinels for surveillance in the most remote areas of the country. Once more, education on how to recognize and report suspect cases in these particular locations is critical to ensure maximal response efficiency. The primary healthcare system must therefore work in close collaboration with the community, on the forefront of surveillance. Indeed, EVD emerges from its natural niche without any known precursor signs, and outbreaks have always surprised local communities. Continuous active surveillance is needed and must be organized by specialized centers joined in local, regional, and international networks. Surveillance centers must be connected to public health systems and also to research teams for increased understanding and awareness to permit identification and implementation of rapid intervention strategies.

As viruses do not heed borders, a global approach is absolutely necessary. The medical community has recognized this concept, one of linking an international coordinated approach to pathogen emergence.^11^ Viruses easily transgress administrative borders via travelers who have yet to present with or recognize symptoms. Therefore, border regions must be permeable to both regional and international healthcare systems. The healthcare worker must be able to effectively intervene in a manner that is not constrained by administrative challenges between countries, fostering communication and exchange beyond the country’s limits. Concurrently, sanitary control measures and surveillance must be particularly enforced at border checkpoints to reduce the risk of infected persons crossing over. Indeed, these checkpoints are needed to screen individuals for signs and symptoms, while also providing opportunity to inform travelers of health risks.

Wide-scale communication to the public, community counseling, and social mobilization programs need to be enacted at the very beginning of a suspected epidemic. Often, insufficient communication and dissemination of inaccurate information are significant impediments to public health initiatives. Populations at risk in Sierra Leone, Guinea, and Liberia are extremely varied and include many different age groups, religious affiliations, as well as social and ethnic backgrounds. These factors must be considered collectively when tailoring communications for efficient dissemination of high-impact messages to targeted populations. The role of social and human sciences is ever more important to inform in real-time, to teach those at risk how to protect themselves, breaking the cycle of transmission through transformative understanding that uproots traditional practices for the betterment of the population.

Clearly a deep understanding and keen recognition of the early signs portending to an emerging outbreak are essential to rapidly identify and isolate cases and contacts and interrupt and quell the dynamic of exponential viral transmission. Vulnerable populations must be clearly identified and fully understood. Healthcare systems must be immediately alerted and communication through regional networks must be enacted in a manner that permits quick adaptation to the rapidly evolving situation. The current outbreak has defied borders and is now a matter of concern for the international community, increasing the complexity of transmission dynamics and disease management, risking socio-political destabilization. Sensationalistic communication fueling fear, coupled with ignorance, has galvanized an unprecedented “journey” of the Ebola virus from Monrovia to Lagos to Dallas. In the aftermath of the outbreak it will be of critical importance to carefully consider the gaps, the missteps, which have permitted this emergence and ultimately, through technological advancement and lessons learned, prevention and control will certainly improve.

## Author Contributions

NW, SHK, MC, VL: Actively worked on generating clinical and field data and processed the data; JB, LM, MG, DK: Actively worked on generating field data and processing data in a common data base. JB, LM, MG, DK, MSKS, IAKG, JLBM, LK, KK, WR: Largely generated complex and complete field data, contact tracing and interviews patient and families on the field; AG, JS, ML, MY, MV, AJ, JF, MG: Coordinate all team from the laboratory, to the field epidemiology and clinics. NW, MS, JPG, TV, MG: developed the concept, did the data analysis, produce the figures and wrote the manuscript.

## Competing Interests Statement

All of the authors listed read the present version of the manuscript and do not have any conflict of interest with respect to the manuscript.

## Figures and Tables

**Figure S2. Primary site of Ebola Virus Disease (EVD) emergence featuring the Kailahun District, Eastern Province of Sierra Leone.**
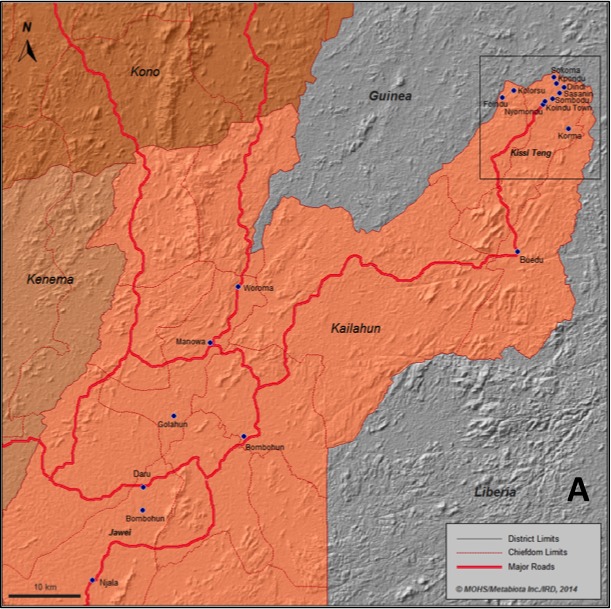
Square = northern tip of Kailahun District were EVD emerged in country; Blue solid circle = Villages with laboratory confirmed case of EVD (i.e. epidemic chain) during the 3 week time period after the first EVD laboratory confirmed case in country.

**Figure S2B. Primary site of Ebola Virus Disease (EVD) emergence featuring the Kailahun District, Eastern Province of Sierra Leone.**
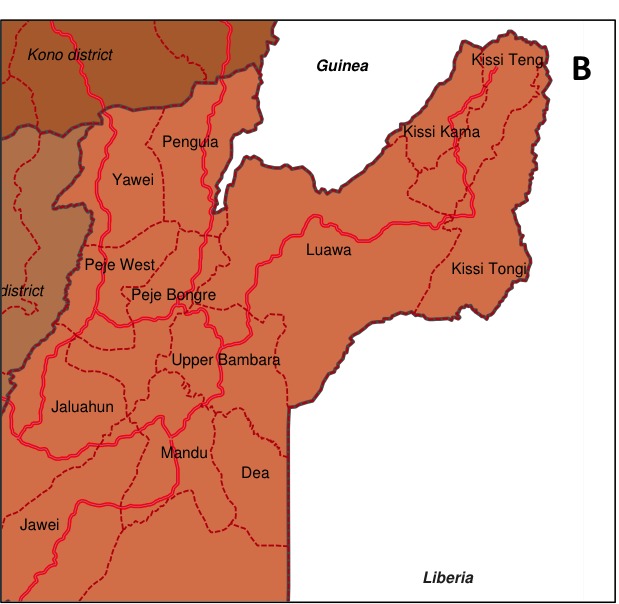
Map of Northern Kailahun District featuring the chiefdoms and their border (red dotted line).
